# Correction: Recurrence pattern predicts aneurysm rupture after coil embolization

**DOI:** 10.1371/journal.pone.0305497

**Published:** 2024-06-11

**Authors:** Iku Nambu, Kouichi Misaki, Takehiro Uno, Akifumi Yoshikawa, Naoyuki Uchiyama, Masanao Mohri, Mitsutoshi Nakada

In Fig 1, the alphabet is incorrect. Please see the correct Fig 1 here.

**Fig 1:**
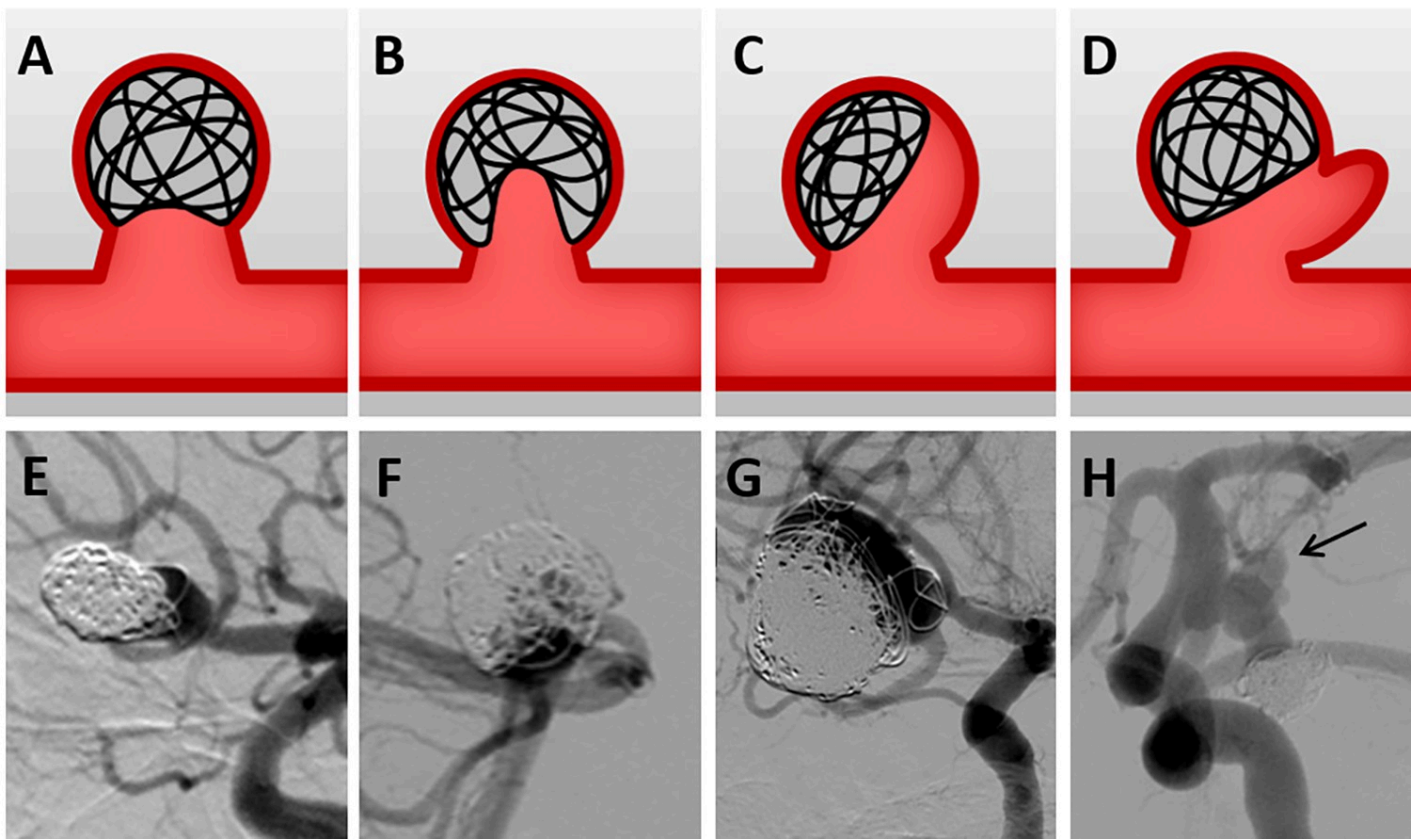
Classification of aneurysm recurrence patterns. (A and E) Type Ⅰ, enlargement of aneurysm neck. (B and F) Type Ⅱ, recurrent cavity within the coil mass. (C and G) Type Ⅲ, recurrent cavity along the aneurysm wall. (D and H) Type Ⅳ, formation of a daughter sac. Black arrow indicates a daughter sac.
