# Supplementary material for: Recurrence pattern predicts aneurysm rupture after coil embolization
Source: PLoS One. 2022 Sep 21;17(9):e0261996. doi: 10.1371/journal.pone.0261996 (PMC9491535; doi:10.1371/journal.pone.0261996)
Supplement: Attachment — Submitted filename: Response to Reviewers.docx [file pone.0261996.s002.docx]

February 14, 2021

Stephan Meckel, MD, PhD

Academic Editor

*PLOS ONE*

Dear Editor:

I would like to re-submit an original article for publication in PLOS ONE, titled “Recurrence pattern predicts aneurysm rupture after coil embolization.” The manuscript number is PONE-D-20-27179.

The manuscript has been carefully rechecked and appropriate changes have been made in accordance with the reviewers’ suggestions. The responses to their comments have been prepared and attached herewith.

We thank you and the reviewers for your thoughtful suggestions and insights, which have enriched the manuscript and produced a more balanced and better account of the research. We hope that the revised manuscript is now suitable for publication in your journal.

I look forward to your reply.

Sincerely,

Kouichi Misaki

Department of Neurosurgery, Graduate School of Medical Science, Kanazawa University

13-1 Takara-machi, Kanazawa

Ishikawa 920-8641, Japan

Phone: +81-76-265-2384

Fax: +81-76-234-4262

Email: [misaki@med.kanazawa-u.ac.jp](mailto:namnamnambuiku@med.kanazawa-u.ac.jp)

**Responses to the Reviewers’ Comments**

**Responses to Reviewer #1**

**Comment:** *“This is an interesting paper about the recurrence pattern prediction of aneurysm rupture after coil embolization. However, aneurysm rupture occurred only in three cases. Therefore, the significant of these findings is very low.”*

**Response:** Thank you for taking the time to read our manuscript and reply. Although there were few ruptured aneurysms and statistical evaluation was insufficient, we demonstrated how coiled aneurysms recurred and occurred hemorrhage. I hope this result will be helpful for management of coiled aneurysms.

**Responses to Reviewer #2**

**Comment:** *“Authors should specify the initial treatment of the recurrent aneurysms (simple coiling/balloon or stent assisted coiling etc.) in order to evaluate whether does a relation exist with the recurrence. It would be of some interest tie the recurrence type with the initial technique used to coil the aneurysm.”*

**Response:** Thank you for taking the time to read our manuscript and reply. In the 22 re-treated aneurysms, balloon and stent assisted coiling were performed 18% (4/22) and 9% (2/22), respectively. No significant difference was found compared with no re-treatment group. We have added this results in the results section (page 12) and Table 2 (page 13).

There was no correlation between recurrence type and the balloon/stent assisted coiling. We have added this results in the Table 3 (page 16).

**Responses to Reviewer #3**

*“The authors present a single institution retrospective review of coiled aneurysms with a focus on recurrences, and they propose a grading system to characterize recurrence patterns. While this topic is interesting, I believe that they authors should revise their analysis of this population to make a more meaningful conclusion.”*

**Comment 1:** *“Please state very clearly how recurrence was defined in incompletely-treated aneurysms.”*

**Response:** Thank you for taking the time to read our manuscript and reply. We have added the definition of recurrence in case of adequately-treated aneurysms and incompletely-treated aneurysms, separately. In case of adequately-treated aneurysms, re-treatment was considered if contrast filling was noted in a cavity occupying > 20% of the volume of original aneurysm. In case of incompletely-treated aneurysm, re-treatment was considered if contrast filling was further increased (page 8, line 124-126).

**Comment 2:** *“It is nice that you focus on the three cases that homorrhaged as this is ultimately the event that we would like to avoid in coiled aneurysm patients. Please report as much as possible regarding the results of interval scans in these patients so that we can better understand the natural history of aneurysm recurrence.”*

**Response:** According to your suggestion, we have added more detailed follow-up about 3 recurrent hemorrhage aneurysms. In case #1, the basilar tip aneurysm with incomplete occlusion, no change was observed on MRA 1 month after coiling, but recurrence with Type III was observed on MRA 4 months after coiling. Hemorrhage occurred 2 months after final MRI (page 21, line 298-301). In case #2 of PcomA aneurysm, follow-up annual MRA showed the remnant neck had gradually increased in size until 6^th^ year, and new sac was formed at 7^th^ year after coiling. Although re-treatment was scheduled, re-hemorrhage occurred 2 months after final MRA (page 21, line 305-308). In case #3 of basilar tip aneurysm, the aneurysm recurred with neck enlargement, and a new sac was formed at 7^th^ year after treatment. Although re-treatment was scheduled, re-hemorrhage occurred 2 months after the final MRA (page 21, line 309-314).

**Comment 3:** *“Along similar lines, reporting the results of interval scans for all of the recurrent aneurysms would be valuable. The occurrence and rate of aneurysm growth and/or coil compaction may be just as important a finding as the pattern of recurrence.”*

**Response:** According to your suggestion, we have added the results of interval scans and rate of aneurysm growth and/or coil compaction. In the Type I recurrence aneurysms (n=7), 4 patients had major recurrence 6 months after coiling. Another 3 patients had minor recurrence 6 months after coiling, and developed major recurrence 12 months after coiling (page 18, line 248-250, and Table 3). In the Type II recurrence aneurysms (n=4), 2 patients had major recurrence 6 months after coiling. Another 2 patients had major recurrence 12 and 18 months after coiling, respectively (page 18, line 253-255, and Table 3). In the Type III recurrence aneurysms (n=9), 8 patients had major recurrence within 6 months after coiling (page 18, line 259-260, and Table 3).

In the Type I recurrence group, coil compaction without aneurysm growth was the most frequent. In the Type II and III recurrence group, almost of all aneurysms revealed coil compaction with aneurysm growth (Table 3). The definition of aneurysm growth and coil compaction were added in the Methods (page 9, line 129-131).

**Comment 4:** *“Although risk factors for aneurysm recurrence after coiling have been previously described, this manuscript would be much stronger if you performed a multivariate analysis of risk factors associated with recurrence (both overall and for each recurrence pattern) and hemorrhage.”*

**Response:** According to your suggestion, we evaluated risk factors associated with re-treatment after coil embolization using univariate and multivariate analysis. In the re-treatment group, the proportion of patients with ruptured aneurysms was significantly higher, maximum size and neck width were significantly larger, the proportion of BA and MRRC class IIIb were significantly higher, and VER was significantly lower than in the no re-treatment group. In multivariate analysis, rupture status and maximum diameter were statistically significant (page12, line 186-195, and Table 2).

We also evaluated the risk factors associated with each recurrence patterns. Larger size, wider neck, and lower VER were associated with Type II recurrence. Ruptured status, larger size, wider neck, MRRC class IIIb, and lower VER were associated with Type III recurrence (page 16-18, Table 3). The number of each recurrence patterns and hemorrhage were small, multivariate analysis could not be performed.

**Comment 5:** *“Please discuss whether you think there is a continuum between your recurrence patterns.”*

**Response:** I think there is a continuum between recurrence patterns. In this study, Type I recurrence aneurysms developed to Type IV recurrence. Type I or II recurrence aneurysms can progress into Type III recurrence, but this progression did not occur in this study. The reason why, re-treatments were performed before the progression.

**Comment 6:** *“The CFD data is interesting but adds little to this study since it was only used for two examples. If possible, this manuscript would be significantly stronger with a more complete CFD analysis of recurrent aneurysms.”*

**Response:** According to your suggestion, we conducted additional CFD analysis for recurrent aneurysms. We previously reported that high pressure at the coil surface was the strongest predictor for posterior communicating artery aneurysm recurrence after coiling. According to the previous study, we conducted CFD analysis for recurrent posterior communicating artery aneurysms (n=5), and evaluated pressure difference at the coil surface. In this study, the value of pressure difference at the coil surface was 3.44±0.32. This value was higher than reported cut-off value of 2.83, hence high pressure at the coil surface induce recurrence after coiling. We have added the definition of pressure difference in the Method (page 10, line 161-172), the results of pressure difference and illustrative case (page 18, line 263-278), and Discussion (page 24, line 367-374).

**Comment 7:** *“Please include percentages of patients in each recurrence group (rather than just absolute numbers) in the Abstract, Results, and Tables.”*

**Response:** According to the suggestion, we have added the percentage of patients in each recurrence group in the Abstract (page 2, line 32), Results (page 15, line 218-219), and Table 3 (page 16).
