# Supplementary material for: Recurrence pattern predicts aneurysm rupture after coil embolization
Source: PLoS One. 2022 Sep 21;17(9):e0261996. doi: 10.1371/journal.pone.0261996 (PMC9491535; doi:10.1371/journal.pone.0261996)
Supplement: Attachment — Submitted filename: Response to Reviewers.docx [file pone.0261996.s003.docx]

August 3, 2021

Stephan Meckel, MD, PhD

Academic Editor

*PLOS ONE*

Dear Editor:

I would like to re-submit an original article for publication in PLOS ONE, titled “Recurrence pattern predicts aneurysm rupture after coil embolization.” The manuscript number is PONE-D-20-27179.

The manuscript has been carefully rechecked and appropriate changes have been made in accordance with the editor’s suggestions. The responses to the comments have been prepared and attached herewith.

We thank for your thoughtful suggestions and insights, which have enriched the manuscript and produced a more balanced and better account of the research. We hope that the revised manuscript is now suitable for publication in your journal.

I look forward to your reply.

Sincerely,

Kouichi Misaki

Department of Neurosurgery, Graduate School of Medical Science, Kanazawa University

13-1 Takara-machi, Kanazawa

Ishikawa 920-8641, Japan

Phone: +81-76-265-2384

Fax: +81-76-234-4262

Email: [misaki@med.kanazawa-u.ac.jp](mailto:namnamnambuiku@med.kanazawa-u.ac.jp)

**Responses to the Editor’s Comments**

**Comment 1:** *“Please leave the CFD analysis out completely as it has no relevance with only addressing 5 cases.”*

**Response:** Thank you for taking the time to read our manuscript and reply. We have left out the CFD analysis about 5 cases.

**Comment 2:** *“No statistical correction for multiple testing of your dataset has been performed – please contact dedicated statistician to solve this problem – your dataset was tested with many statistical tests for many different questions.”*

**Response:** According to your suggestion, we have added multiple-testing correction. Kruskal-Wallis one-way non-parametric ANOVA was performed for each parameter (maximum diameter, neck width, and volume embolization ratio) for each of the five groups (no re-treatment, recurrence type I, type II, type III, and type IV). Post-hoc pairwise group comparisons were performed using Dunnett’s multiple-testing correction. Each re-treatment group (recurrence type I, II, III, and IV) was compared with the control group of no re-treatment group. We have added these sentences in the Method (page 11, line 174-182), Table 3 (page 16-17), the results (page 18, line 246-259).
