# Supplementary material for: Recurrence pattern predicts aneurysm rupture after coil embolization
Source: PLoS One. 2022 Sep 21;17(9):e0261996. doi: 10.1371/journal.pone.0261996 (PMC9491535; doi:10.1371/journal.pone.0261996)
Supplement: Attachment — Submitted filename: Response to Reviewers.docx [file pone.0261996.s004.docx]

October 6, 2021

Stephan Meckel, MD, PhD

Academic Editor

*PLOS ONE*

Dear Editor:

I would like to re-submit an original article for publication in PLOS ONE, titled “Recurrence pattern predicts aneurysm rupture after coil embolization.” The manuscript number is PONE-D-20-27179.

The manuscript has been carefully rechecked and appropriate changes have been made in accordance with the editor’s suggestions. The responses to the comments have been prepared and attached herewith.

We thank for your thoughtful suggestions and insights, which have enriched the manuscript and produced a more balanced and better account of the research. We hope that the revised manuscript is now suitable for publication in your journal.

I look forward to your reply.

Sincerely,

Kouichi Misaki

Department of Neurosurgery, Graduate School of Medical Science, Kanazawa University

13-1 Takara-machi, Kanazawa

Ishikawa 920-8641, Japan

Phone: +81-76-265-2384

Fax: +81-76-234-4262

Email: [misaki@med.kanazawa-u.ac.jp](mailto:namnamnambuiku@med.kanazawa-u.ac.jp)

**Responses to the Editor’s Comments**

**Comment 1:** *“Thank you for the added statistics with regards to multiple testing correction – any changes to the results?”*

**Response:** Thank you for taking the time to read our manuscript and reply. In the previous results, the maximum diameter and neck width (Type II and III) were significantly larger, and VER (Type II and III) were significantly lower than in the no re-treatment group. In the multiple testing correction, there was no statistically significant difference in the maximum diameter in the Type II.

**Comment 2:** *“CFD should be left out from the paper.”*

**Response:** According to your suggestion, we have left out the CFD analysis from Abstract, Methods, Results, and Discussion. Instead of CFD analysis, we have added some words (page 2; line 35-36, page2; line 38-40, page 23; line 347-348, page 23; line 353-355).
